# Unraveling the complex web of acute kidney injury: pathways, biomarkers, and future directions

**DOI:** 10.1590/2175-8239-JBN-2025-0369en

**Published:** 2026-06-12

**Authors:** Chengxi Zha, Yaya Qi, De Yang, Guoduo Li, Zhijie He, Deping Xu, Jiansheng Li

**Affiliations:** 1 Gansu Provincial Hospital of Traditional Chinese Medicine, Clinical Laboratory, Lanzhou, China.; 2 First Hospital of Lanzhou University, Precision Medicine Experimental Center, Lanzhou, China.; 3 Gansu Provincial Hospital of Traditional Chinese Medicine, Nephrology Department, Lanzhou, China.

**Keywords:** Acute Kidney Injury, Signal Transduction, Early Diagnosis, Personalized Treatment.

## Abstract

Acute kidney injury (AKI), a widespread severe acute condition caused by several factors and characterized by a rapid decline in renal function over a short time, is associated with high incidence and mortality rates worldwide. Investigating AKI signaling pathways is crucial for elucidating its pathogenesis, optimizing diagnostic and therapeutic approaches, and developing novel therapeutic agents. This article comprehensively examines AKI pathophysiological mechanisms, apoptosis-related signaling pathways, and inflammatory response pathways. It analyzes clinical signaling pathways associated with epidemiology, diagnostic technologies, and therapeutic strategies, explores advancements in novel biomarker applications, drug development, and gene editing technologies, and discusses current hot topics and controversies. The study further outlines future research trends, personalized treatment prospects, and potential challenges and opportunities, aiming to provide comprehensive insights into AKI signaling pathway research and lay the foundation for further exploration in related fields.

## INTRODUCTION

Acute kidney injury (AKI) constitutes a complex and multifactorial disorder characterized by a rapid decline in renal function, leading to the accumulation of metabolic waste products and systemic dysregulation^1^. This condition arises from arises from various causes, including ischemia-reperfusion injury, sepsis, exposure to nephrotoxic agents, and hemodynamic instability, and is associated with significant morbidity and mortality^2,3,4^. Despite progress in supportive care, the pathophysiological mechanisms underlying AKI remain only partially understood, particularly regarding the complex signaling pathways involved in cellular injury, inflammation, and maladaptive repair processes. This review comprehensively explores the molecular mechanisms underlying AKI, with an emphasis on the critical signaling pathways that govern the initiation, progression, and recovery of injury, with the goal of identifying potential therapeutic targets.

### Pathological Mechanism and Signaling Pathway of AKI

Ischemia-reperfusion injury is a common cause of AKI. Empirical evidence demonstrates that ischemia-reperfusion exacerbates oxidative stress responses, resulting in the production of substantial quantities of reactive oxygen species (ROS), which damage cellular membranes, proteins, and DNA. This oxidative stress initiates a cascade of intracellular signaling pathways, notably the mitogen-activated protein kinase (MAPK) and nuclear factor kappa-light-chain-enhancer of activated B cells (NF-κB) pathways, culminating in inflammatory responses and apoptosis^5^. In a rat model of renal ischemia-reperfusion, ROS levels in renal tissues increased significantly, accompanied by elevated phosphorylation levels of extracellular signal-regulated kinase (ERK), c-Jun N-terminal kinase (c-JNK), and p38 MAPK within the MAPK pathway. Further research indicates that following renal ischemia-reperfusion injury, serum malondialdehyde (MDA) levels in mice doubled, while blood urea nitrogen (BUN) and creatinine (CREA) levels increased by 5- to 10-fold. Concurrently, the expression levels of nuclear factor erythroid 2-related factor 2 (Nrf2), phosphorylated protein kinase B (p-Akt), heme oxygenase-1 (HO-1), and pro-caspase-3 were reduced in renal tissues, whereas the expression of cleaved caspase-3 was elevated. Administration of Tempol at 50 mg/kg effectively mitigated these alterations, indicating that Tempol attenuates lipid peroxidation and ameliorates renal injury via the PI3K/Akt/Nrf2 signaling pathway^6^. In a murine model of renal ischemia-reperfusion injury, renal levels of progranulin (PGRN) were markedly diminished. In contrast, PGRN-deficient (Grn(–/–)) mice exhibited elevated serum CREA, increased tubular epithelial cell apoptosis, and heightened infiltration of neutrophils and macrophages within the renal interstitium, culminating in exacerbated renal dysfunction. Administration of recombinant human PGRN attenuated hypoxia-induced inflammatory responses and apoptosis in proximal tubule epithelial cells, a process linked to NOD2-mediated immune responses. Notably, both pretreatment and delayed administration of recombinant human PGRN protected wild-type and Grn(-/-) mice against renal ischemia-reperfusion injury or facilitated their recovery, with comparable protective effects observed in cisplatin-induced AKI^7^.

Research has shown that nephrotoxic agents, including cisplatin and contrast agents, can precipitate AKI via distinct signaling pathways. Specifically, cisplatin induces DNA damage within renal tubular epithelial cells, thereby activating the p53 signaling pathway. This pathway modulates the expression of genes involved in cell cycle arrest and apoptosis, culminating in elevated apoptosis rates. In renal tubular epithelial cells exposed to cisplatin, there is an upregulation of p53 protein expression, characterized by increased expression of the pro-apoptotic gene Bax, decreased expression of the anti-apoptotic gene Bcl-2, and heightened activity of apoptotic execution proteins such as caspase-3, ultimately leading to apoptosis^8^. In aging kidneys, diminished expression of α(E)-catenin renders renal tubular epithelial cells more vulnerable to drug-induced AKI. Empirical evidence indicates that cisplatin-induced AKI results in a 5.5-fold increase in Fas expression in C2 cells with stable knockdown of α(E)-catenin, compared to non-targeted control NT3 cells. This is accompanied by increased activation of caspase-8 and -9, reduced Bcl-2 expression, and elevated BID cleavage and cytochrome C release, indicating an amplification of Fas-mediated apoptotic signaling^8^. The NF-κB signaling pathway undergoes significant alterations in cisplatin-induced AKI. On the third day following the administration of cisplatin (25 mg/kg) in mice, manifestations of AKI, acute tubular necrosis (ATN), and apoptosis were observed, along with activation of the NF-κB signaling pathway in renal tissue. Administration of JSH-23 (40 mg/kg), which directly modulates NF-κB transcriptional activity, improved renal function and reduced tubular injury, as indicated by reductions in ATN and serum neutrophil gelatinase-associated lipocalin (NGAL) levels, although it did not significantly impact apoptosis. Further investigations demonstrated that both cisplatin and JSH-23 can up-regulate or down-regulate genes associated with the NF-κB pathway, including IL-10, IFN-γ, and caspase-1^9^.

Additionally, multiple signaling pathways implicated in inflammatory responses are critically involved in the pathogenesis of AKI. Inflammatory mediators such as tumor necrosis factor-α (TNF-α) and interleukin-1β (IL-1β) were found to be up-regulated, with a marked increase in apoptosis of renal tubular epithelial cells^5^. Research has further shown that Toll-like receptor (TLR)-mediated inflammatory responses, including TLR-2, TLR-4, nuclear NF-κB p65, phosphorylated ASK1, phosphorylated TRAF2, IL-1β, IL-6, and IL-18, are elevated in this context^10^.

In sepsis-induced AKI, glycyrrhizin (GA) has several therapeutic effects, including the mitigation of pathological renal alterations, reduction of BUN and CREA levels, and increased survival rates in rat models. Furthermore, GA inhibits the production of pro-inflammatory cytokines associated with the NF-κB signaling pathway, specifically TNF-α, IL-1β, and IL-6. It also decreases the generation of nitric oxide (NO) and prostaglandin E2 (PGE2) in renal tissues, while promoting the expression of inducible nitric oxide synthase (iNOS) and cyclooxygenase-2 (COX-2), thereby reducing apoptosis in renal cells^11^.

Furthermore, the angiogenesis-Tie2 signaling pathway plays a role in the pathophysiological mechanisms of AKI. Ischemic acute kidney injury (IR-AKI) is estimated to affect 2-7% of hospitalized patients, and activation of the angiogenesis-Tie2 tyrosine kinase signaling pathway has been shown to ameliorate ischemia-reperfusion injury (IRI). Research indicates that in IR-AKI mouse models, the phosphatase VE-PTP, which acts as a negative regulator of the angiogenesis-Tie2 pathway, is upregulated in renal endothelial cells following ischemia, whereas its gene knockout confers protection against IR-AKI. Simultaneously, injection of the novel angiogenesis analog Hepta-ANG1 effectively activates Tie2 and protects renal function in mice. Single-cell RNA sequencing analysis identified endothelium-specific gene signatures and the emergence of a new glomerular endothelial subpopulation associated with improved renal function^12^. Table 1 shows a summary of the types, mechanisms, and pathways of AKI.

**Table 1:** The types, mechanisms and pathways of AKI

Type	Pathogenesis	Signaling pathways	Critical molecules
Prerenal AKI	1. Systemic hypoperfusion → decreased renal blood flow (RBF) → reduced glomerular filtration rate (GFR) 2. Activation of compensatory mechanisms 3. Persistent hypoperfusion progresses to intrinsic AKI	1. RAAS Pathway Sympathetic 2. Nervous System Signaling	Renin, Angiotensin II, Aldosterone, Norepinephrine, Antidiuretic Hormone (ADH)
Intrinsic AKI	1. **Ischemic injury**: Mitochondrial dysfunction, ATP depletion, cell calcium overload 2. **Nephrotoxicity**: Direct cellular damage, oxidative stress, inflammatory infiltration 3. **Tubular obstruction**: Cast formation, cellular debris accumulation	1. **Oxidative Stress Pathway**: Nrf2/HO-1 Pathway 2. **Inflammatory Pathway**: NF-κB Pathway, JAK/STAT Pathway 3. **Apoptosis & Necroptosis Pathway**: MAPK Pathway, PI3K/Akt Pathway, Caspase Cascade 4. **Fibrosis-Related Pathway**: TGF-β/Smad Pathway	1. **Oxidative stress**: Reactive Oxygen Species (ROS), Nuclear factor erythroid 2-related factor 2 (Nrf2), Heme Oxygenase-1 (HO-1) 2. **Inflammation**: Nuclear Factor-kappa B (NF-κB), Tumor Necrosis Factor-α (TNF-α), Interleukins (IL-1β, IL-6, IL-18) 3. **Cell death**: Caspase-3, Caspase-8, Receptor-interacting protein kinase 1/3 (RIPK1/3) 4. **Fibrosis & repair**: Transforming Growth Factor-β (TGF-β), Smad2/3, Fibronectin, Collagen Type I
Postrenal AKI	Urinary tract obstruction → increased intratubular pressure → decreased effective filtration pressure → reduced GFRPersistent obstruction induces renal interstitial inflammation, tubular atrophy and renal fibrosis	1. NF-κB Inflammatory PathwayTGF-β 2. Smad Fibrosis Pathway 3. MAPK Pathway	NF-κB, TNF-α, IL-6, TGF-β, Smad2/3, Tissue Inhibitor of Metalloproteinase (TIMP-1)

### Signaling Pathway Markers for AKI Diagnosis

Current clinical diagnostic methods for AKI, such as serum CREA levels and urine output measurements, are inadequate for early detection. Consequently, the development of novel biomarkers based on signaling pathways has become essential for enhancing diagnostic precision^13^. Neutrophil gelatinase-associated lipocalin (NGAL) has emerged as a promising biomarker for AKI. Research indicates that plasma NGAL levels are correlated with the risk of developing chronic kidney disease (CKD) in the general population. In a prospective cohort study involving 4,660 individuals without CKD followed over a period of 8.3 years, 467 participants developed new-onset CKD. Plasma NGAL concentrations were significantly associated with an increased risk of new-onset CKD (hazard ratio [HR] 1.35, 95% confidence interval [CI]: 1.11–1.63, p = 0.002), even after adjusting for confounding variables (HR 1.37, 95% CI: 1.09–1.73, p = 0.007). However, this association was no longer significant after adjusting for baseline estimated glomerular filtration rate (eGFR) (HR 1.09, 95% CI: 0.86-1.37, p = 0.490)^14^.

In patients with COVID-19, research has identified several biomarkers associated with signaling pathways that hold promise for the early diagnosis of AKI. A comprehensive review of the literature indicates that markers of proximal tubule injury and early-stage ATP metabolites, which reflect energy metabolism disorders, may serve as vital clinical indicators for the detection of AKI^15^. Simultaneously, advancements in novel detection technologies for molecular urine biomarkers (MURs), such as gamma-glutamyl transferase (GGT), alanine aminotransferase (AAP), and N-acetyl-β-D-glucosaminidase (NAG), are ongoing. In mouse models of drug-induced AKI and CKD, MURs not only facilitate earlier detection of nephrotoxicity compared to traditional clinical diagnostic methods but also exhibit greater diagnostic accuracy, offering a novel approach for the early diagnosis of AKI^16^. Table 2 provides a summary of biomarkers for AKI.

**Table 2:** Summary of AKI diagnostic biomarkers

Biomarkers	Advantages	Disadvantages
Screa	Easy to detect, low cost and wide availability	Lag, poor tissue specificity
BUN	Easy to detect, low cost and wide availability	Lag, poor specificity
urine output (UO)	Real-time, non-invasive	Poor specificity
NGAL	High sensitivity and specificity	Poor tissue specificity
Kidney Injury Molecule-1 (KIM-1)	High tissue specificity	Less sensitive, not suitable for routine screening
NAG	High sensitivity	Poor specificity
TIMP-2·IGFBP7	Early, high specificity	High cost, not suitable for routine screening
**IL-18**	High sensitivity	Poor specificity

### Signaling Pathway Targets for AKI Treatment

The management of AKI continues to face significant challenges; however, therapeutic strategies targeting specific signaling pathways present a promising approach for enhancing AKI outcomes^17^. Research indicates that zingerone can mitigate lipopolysaccharide (LPS)-induced AKI by inhibiting the TLR4/NF-κB inflammatory signaling pathway. In murine models of LPS-induced AKI, zingerone administration resulted in a dose-dependent reduction of elevated BUN, CREA, and inflammatory cytokines such as TNF-α, IL-6, and IL-1β, alongside a decrease in renal histopathological alterations. These findings suggest that zingerone confers protective effects against LPS-induced AKI through the inhibition of this signaling pathway^18^.

In renal ischemia-reperfusion injury, enhancer of zeste homolog 2 (EZH2) is pivotal in AKI pathogenesis by modulating the p38 signaling pathway. Administration of the EZH2 selective inhibitor 3-deazaneplanocin A (DZNeP) in mice ameliorated renal dysfunction and tubular damage post-ischemia-reperfusion, attenuated apoptosis and caspase-3 activation, and suppressed the recruitment of CD3+ T cells and F4/80+ cells, as well as the production of inflammatory mediators, including TNF-α, monocyte chemoattractant protein-1 (MCP-1), IL-6, and IL-18. In cellular experiments, treatment with DZNeP or knockdown of EZH2 resulted in apoptosis reduction in HK-2 cells subjected to hypoxia-reoxygenation. This finding indicates that targeting the EZH2/p38 signaling pathway may represent a novel strategy for safeguarding renal tissues against ischemia-reperfusion-induced AKI^19^.

Concurrently, certain pharmacological agents exhibit therapeutic potential in AKI by modulating distinct signaling pathways. For example, lithium administration has been shown to significantly enhance renal function in rat models, as evidenced by reduced kidney injury scores, decreased levels of creatine phosphokinase (CPK), diminished macrophage infiltration, and lowered renal expression of NF-κB and caspases. Additionally, lithium treatment increases levels of manganese superoxide dismutase (MnSOD), an antioxidant enzyme. These therapeutic effects are primarily attributed to the inhibition of glycogen synthase kinase 3β (GSK3β), thereby ameliorating AKI associated with rhabdomyolysis^20^.

### Hotspots in the Research of AKI Signaling Pathway

The Bone Morphogenetic Protein 7 (BMP-7) signaling pathway has been a major area of research focus for an extended period. BMP-7 plays a critical role in kidney development and injury repair, with its therapeutic potential in models of chronic kidney disease being acknowledged nearly two decades ago. Recent investigations have elucidated that BMP-7 can mitigate kidney damage in AKI through various mechanisms, including the regulation of extracellular matrix metabolism, suppression of inflammatory responses, and induction of apoptosis. Animal studies have demonstrated that the administration of recombinant BMP-7 enhances renal function and reduces pathological damage, suggesting a promising biological therapeutic strategy for AKI treatment^21^.

Signaling pathways related to non-apoptotic cell death remain a pivotal research focus. In sepsis-induced AKI, receptor-interacting protein kinase 3 (RIPK3) has been shown to exacerbate renal tubular injury by promoting necroptosis, oxidative stress, and mitochondrial dysfunction. Further research has identified RIPK3 and NADPH oxidase-4 (NOX4) as critical factors in renal tubule injury in vivo, thereby offering novel therapeutic targets for AKI^22^.

Transcriptomic analyses have yielded novel insights into the signaling pathways associated with AKI. Through the transcriptomic profiling of damaged renal tubular epithelial cells, researchers have identified multiple signaling pathways and gene regulatory networks that play roles in epithelial plasticity, tissue repair, and fibrosis. The study demonstrated that injured renal tubular epithelial cells attempt to repair themselves by re-expressing certain nephrogenic genes; however, this process deviates from normal developmental pathways, resulting in a unique regenerative response mechanism. These findings advance the understanding of the molecular mechanisms underlying AKI and provide a theoretical foundation for the development of innovative therapeutic strategies^23^. By employing techniques such as weighted gene co-expression network analysis (WGCNA), researchers have identified critical miRNA-mRNA networks associated with AKI. In studies focusing on post-transplant AKI, WGCNA analysis of two datasets (GSE53771 and GSE53769) revealed numerous mRNA and miRNA interactions. Further analysis using miRDIP v4.1 predicted key miRNA-mRNA interaction modules, thereby constructing a comprehensive regulatory network. In particular, nodes such as miR-203a-3p, miR-205-5p, and ERBB4 demonstrated significant connectivity, indicating their critical roles in the development of post-transplant AKI. Concurrently, analyses using the Gene Ontology (GO) and Kyoto Encyclopedia of Genes and Genomes (KEGG) pathways revealed that this network predominantly encompasses kidney-related functions and signaling pathways, including PI3K-Akt, HIF-1, Ras, and MAPK. These findings offer essential insights for the identification of novel biomarkers or therapeutic agents, thereby enhancing early prediction and intervention strategies for AKI^24^.

Biomarkers associated with DNA methylation have garnered increasing scholarly interest. Through the analysis of whole blood DNA methylation in participants from the Taiwan Biobank, researchers have identified specific genes and signaling pathways linked to renal aging and deteriorating kidney function. Among the 1,587 participants studied, 187 demonstrated accelerated rates of eGFR decline. By comparing methylation patterns among participants with different eGFR decline rates and age groups, researchers identified commonly hypermethylated genes, such as DNMT3A and GGACT, as well as hypomethylated genes, including ARL6IP5, CYB5D1, BCL6, RPRD2, ZNF451, and MIAT. Furthermore, the methylation status of signaling pathways, such as autophagy, p38 MAPK, and sirtuins, was found to be associated with aging and renal dysfunction. These findings contribute to the development of novel biomarkers for identifying high-risk populations and offer insights into potential therapeutic targets^25^.

In renal fibrosis research, STAT3 is crucial. Studies using Foxd1-mediated Stat3 knockout mice, CRISPR, and STAT3 inhibitors revealed that STAT3 phosphorylation occurs in renal tubular epithelial cells during AKI and extends to interstitial cells in fibrosis. Foxd1-mediated Stat3 deficiency protected mice from folic acid- and aristolochic acid-induced fibrosis. STAT3 enhances inflammatory responses and pericyte differentiation into myofibroblasts, promoting migration and fibrotic signaling in genome-edited pericyte-like cells. Inhibition of STAT3 reduces cell detachment, migration, and fibrotic signaling. STAT3 also binds to the Collagen1a1 promoter in mouse kidneys and cells. This study identifies STAT3 as a novel promoter of renal fibrosis, suggesting it as a potential gene therapy target^26^.

Moreover, LRP5, a multifunctional transmembrane co-receptor, plays a pivotal role in AKI. It primarily operates through the Wnt/β-catenin signaling pathway, while also modulating renal function via non-classical pathways such as AKT/P21 and TGF-β/Smad. Although LRP5 demonstrates protective effects in the context of AKI, it contributes to disease progression in chronic pathological conditions, including renal tubulointerstitial fibrosis, polycystic kidney disease, and atherosclerosis, by activating fibrotic and inflammatory pathways. Utilizing CRISPR/Cas9 knockout and other gene-editing technologies, researchers can conduct comprehensive investigations into the mechanisms of LRP5 in AKI and related diseases, thereby providing a theoretical foundation for the development of LRP5-based therapeutic strategies^27^.

### Controversial Points in the Study of AKI Signaling Pathways

In the investigation of the AKI signaling pathway, significant advancements have been achieved; however, several contentious issues persist, necessitating further research^8^. The mechanisms of cell death and their associated signaling pathways in AKI remain subjects of debate. For example, research on aging kidneys, which are more susceptible to drug-induced AKI, has demonstrated that diminished expression of α(E)-catenin enhances the Fas-mediated apoptosis pathway. Nevertheless, the precise roles and interactions of other cell death mechanisms, such as necroptosis and ferroptosis, in this context are not yet fully understood. Some studies indicate that necroptosis may act synergistically with apoptosis to intensify renal injury under certain conditions, whereas others suggest that these processes might operate independently at various stages or in response to different stimuli^8^.

The regulatory mechanisms governing inflammatory signaling pathways are also still under debate. The NF-κB signaling pathway serves as a pertinent example, with its activation and inhibition exhibiting variable effects on kidney injury across different AKI models and studies. In some investigations, inhibition of NF-κB signaling has been shown to mitigate inflammation and renal damage. Conversely, activation of this pathway appears to contribute to kidney repair processes. For example, during the early stages of renal ischemia-reperfusion injury, moderate activation of NF-κB may facilitate the initiation of immune responses necessary for the elimination of damaged cells and pathogens. However, excessive activation can result in uncontrolled inflammation and exacerbated tissue damage. Therefore, precise regulation of the NF-κB signaling pathway is essential to achieve optimal therapeutic outcomes, necessitating further investigation^9^.

Moreover, the interactions among various signaling pathways and their dynamic alterations throughout the different stages of AKI remain a subject of debate. Notably, the PI3K-Akt and mTOR signaling pathways are integral to AKI, demonstrating intricate mutual regulatory relationships. Despite this, the precise regulatory mechanisms and their predominant roles across distinct AKI phases, such as the injury and repair phases, are not yet fully understood. Some studies suggest that the PI3K-Akt pathway is primarily involved in cell survival and anti-apoptotic processes during the early injury phase, whereas the mTOR pathway may play a more pivotal role in cell proliferation and tissue repair during the repair phase. Nonetheless, these findings necessitate further investigation to be validated and refined^28^.

### Future Prospects of AKI Signaling Pathways

Research on the signaling pathways of AKI is expected to undergo multifaceted advancements, potentially leading to significant breakthroughs in understanding its pathogenesis and developing effective therapeutic strategies^29^. The role of H2S in renal physiology and pathology warrants further exploration. As a gaseous signaling molecule, H2S plays a regulatory role in key renal physiological processes, including glomerular filtration and sodium reabsorption. In renal diseases, the effects of H2S are complex and context-dependent. For instance, in conditions such as ischemia-reperfusion injury and diabetic nephropathy, H2S has been shown to ameliorate renal damage. Conversely, its role in cisplatin-induced nephrotoxicity remains to be fully elucidated. Future research is expected to comprehensively examine H2S’s influence on renal physiology, elucidate its mechanisms in inflammation- and toxicity-related kidney diseases, assess its potential as a therapeutic target in specific renal disorders, and develop H2S-based therapeutic interventions^29^.

Research on lipid metabolism is essential not only for energy provision in the kidneys but also for the formation of renal biofilms and the development of the renal microenvironment. Metabolites from lipid metabolism significantly influence cellular processes, including proliferation, differentiation, and apoptosis, through signal transduction pathways. Although current research predominantly addresses lipid metabolism abnormalities in CKD, there are relatively few studies on lipid metabolism disorders associated with AKI. Future research could investigate lipid metabolites as potential biomarkers for early diagnosis and classification of AKI and examine the impact of regulating lipid metabolism on AKI progression. Such efforts may unveil novel therapeutic targets and intervention strategies for the management of AKI^30^.

Concurrently, the ongoing progress in single-cell transcriptomics and multi-omics technologies has enhanced our ability to accurately delineate alterations in signaling pathways across diverse cell types during AKI. Through the application of single-cell transcriptomic analysis on renal tissues, researchers can achieve a more profound understanding of the distinct activation patterns of signaling pathways within various cell populations, including renal tubular epithelial cells, endothelial cells, and immune cells, during AKI. This approach also elucidates the mechanisms of intercellular signaling. Such insights are pivotal for unraveling the intricate pathogenesis of AKI and lay the groundwork for the development of precision therapeutic strategies that target specific cell types and signaling pathways^31^.

**Figure 1:**
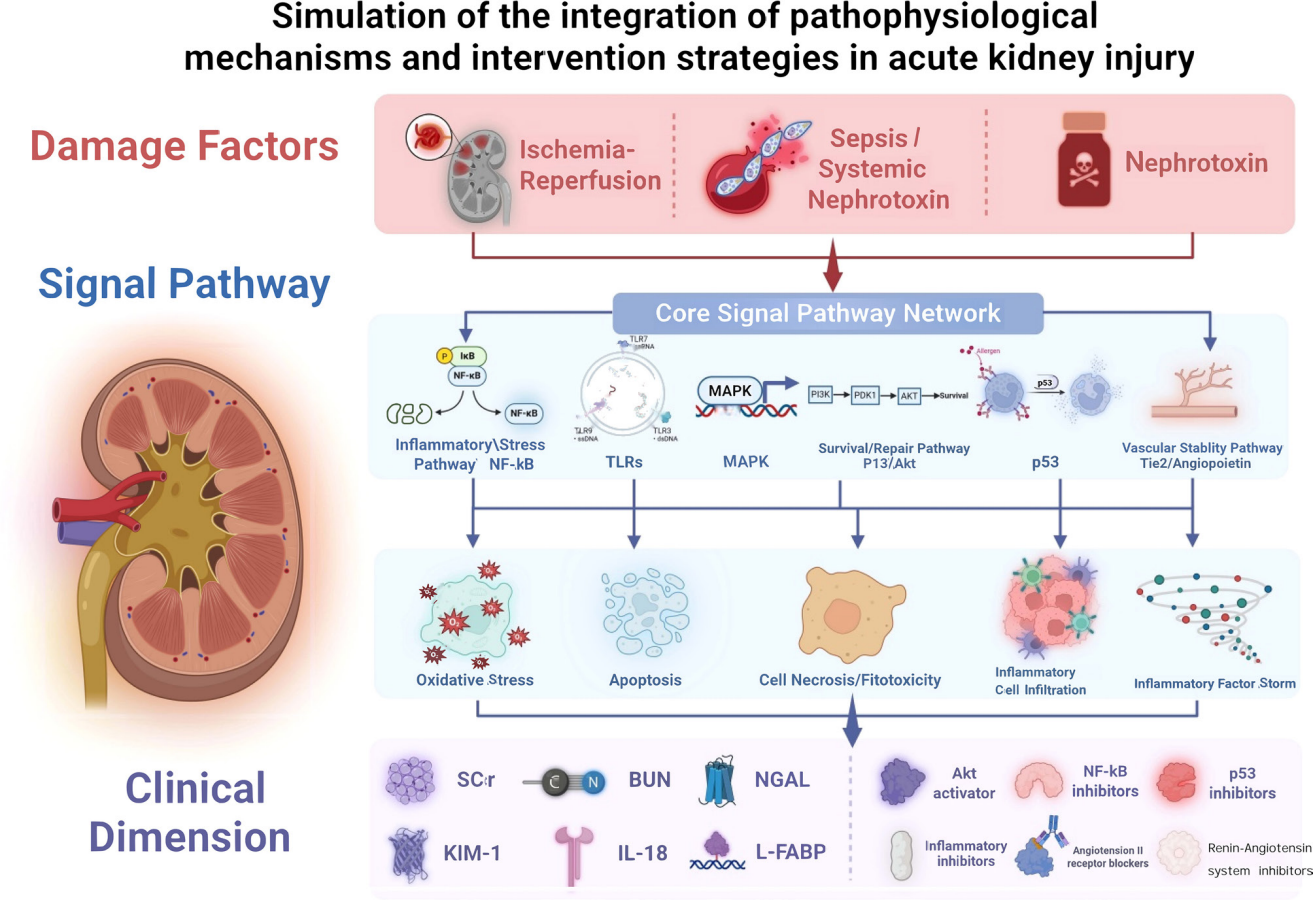
Description of multiple interconnected signaling pathways involved in AKI pathogenesis.

Research on AKI signaling pathways encounters numerous challenges, yet it also presents significant opportunities for ongoing advancements in the field^32^. The intricate pathogenesis of AKI involves interactions among multiple signaling pathways and various cell types, complicating the comprehensive understanding of its pathological processes (Figure 1). In AKI induced by ischemia-reperfusion injury, inflammation-related signaling pathways are activated, and alterations also occur in pathways associated with cellular processes such as apoptosis and autophagy. These signaling pathways have complex inter-regulatory relationships, making the comprehensive and precise analysis of their dynamic changes and interactions a significant challenge^32^. Translating fundamental research findings into clinical applications remains a difficult. Certain signaling pathway inhibitors or activators that exhibit promising renal protective effects in animal models often fail to achieve the expected results in human trials, primarily due to pharmacokinetic and safety concerns. Enhancing the efficiency of clinical translation of basic research findings is an urgent issue that necessitates attention. Nonetheless, research on AKI signaling pathways offers numerous opportunities. The advent of advanced technologies, such as gene editing (e.g., CRISPR/Cas9), single-cell sequencing, and high-resolution imaging, has equipped researchers with powerful tools for conducting in-depth investigations into AKI signaling pathways. Gene editing technologies facilitate precise exploration of the roles of specific genes within these pathways, while single-cell sequencing elucidates pathway alterations across different cell types during AKI. Furthermore, high-resolution imaging allows for the capture of real-time molecular dynamics within renal tissues. Collectively, these technological advancements are set to revolutionize research on AKI signaling pathways^26^.

## Data Availability

No new data were generated or analyzed in this study.
