# Supplementary material for: Unraveling the complex web of acute kidney injury: pathways, biomarkers, and future directions
Source: J Bras Nefrol. 2026 Jun 12;48(3):e20250369. doi: 10.1590/2175-8239-JBN-2025-0369en (PMC13267843; doi:10.1590/2175-8239-JBN-2025-0369en)
Supplement: Translation

# Translation: Desvendando a complexidade da injúria renal aguda: vias, biomarcadores e perspectivas futuras

**DOI:** 10.1590/2175-8239-JBN-2025-0369pt

**Authors:** Chengxi Zha, Yaya Qi, De Yang, Guoduo Li, Zhijie He, Deping Xu, Jiansheng Li

**Affiliations:** 1 Gansu Provincial Hospital of Traditional Chinese Medicine, Clinical Laboratory, Lanzhou, China.; 2 First Hospital of Lanzhou University, Precision Medicine Experimental Center, Lanzhou, China.; 3 Gansu Provincial Hospital of Traditional Chinese Medicine, Nephrology Department, Lanzhou, China.

**Keywords:** Injúria Renal Aguda, Transdução de Sinais, Diagnóstico Precoce, Tratamento Personalizado.

## Abstract

A injúria renal aguda (IRA), uma condição aguda grave e amplamente disseminada, causada por diversos fatores e caracterizada por um rápido declínio da função renal em curto período de tempo, está associada a elevadas taxas de incidência e mortalidade em todo o mundo. A investigação das vias de sinalização da IRA é crucial para elucidar sua patogênese, otimizar abordagens diagnósticas e terapêuticas e desenvolver novos agentes terapêuticos. Este artigo examina de forma abrangente os mecanismos fisiopatológicos da IRA, as vias de sinalização relacionadas à apoptose e as vias de resposta inflamatória. Analisa, ainda, as vias de sinalização clínicas associadas à epidemiologia, às tecnologias diagnósticas e às estratégias terapêuticas, explora os avanços na aplicação de novos biomarcadores, no desenvolvimento de medicamentos e nas tecnologias de edição genômica, além de discutir temas atuais polêmicos e controvérsias. O estudo também delineia tendências futuras de pesquisa, perspectivas de tratamento personalizado e potenciais desafios e oportunidades, com o objetivo de fornecer uma visão abrangente da pesquisa sobre as vias de sinalização da IRA e estabelecer as bases para futuras investigações em áreas relacionadas.

## Introdução

A injúria renal aguda (IRA) constitui uma doença complexa e multifatorial caracterizada por um rápido declínio da função renal, levando ao acúmulo de resíduos metabólicos e à desregulação sistêmica^1^. Essa condição decorre de diversas causas, incluindo lesão por isquemia-reperfusão, sepse, exposição a agentes nefrotóxicos e instabilidade hemodinâmica, estando associada a morbidade e mortalidade significativas^2,3,4^. Apesar dos avanços nos cuidados de suporte, os mecanismos fisiopatológicos subjacentes à IRA permanecem apenas parcialmente compreendidos, principalmente no que se refere às complexas vias de sinalização envolvidas na lesão celular, na inflamação e nos processos de reparo mal adaptativo. Esta revisão explora de forma abrangente os mecanismos moleculares subjacentes à IRA, com ênfase nas vias de sinalização críticas que regem o início, a progressão e a recuperação da lesão, com o objetivo de identificar potenciais alvos terapêuticos.

## Mecanismo Patológico e via de Sinalização da IRA

A lesão por isquemia-reperfusão é uma causa comum de IRA. Evidências empíricas demonstram que a isquemia-reperfusão agrava as respostas ao estresse oxidativo, resultando na produção de quantidades substanciais de espécies reativas de oxigênio (ROS), que danificam as membranas celulares, as proteínas e o DNA. Esse estresse oxidativo inicia uma cascata de vias de sinalização intracelulares, notadamente as vias da proteína quinase ativada por mitógeno (MAPK) e do fator nuclear kappa B (NF-κB), culminando em respostas inflamatórias e apoptose^5^. Em um modelo de isquemia-reperfusão renal em ratos, os níveis de ROS nos tecidos renais aumentaram significativamente, acompanhados por níveis elevados de fosforilação da quinase regulada por sinal extracelular (ERK), da quinase N-terminal c-Jun (c-JNK) e da p38 MAPK, no contexto da via MAPK. Pesquisas adicionais indicam que, após lesão por isquemia-reperfusão renal, os níveis séricos de malondialdeído (MDA) em camundongos duplicaram, enquanto os níveis de nitrogênio ureico no sangue (BUN) e creatinina (CREA) aumentaram entre 5 e 10 vezes. Ao mesmo tempo, os níveis de expressão do fator nuclear eritroide 2 relacionado ao fator 2 (Nrf2), da proteína quinase B fosforilada (p-Akt), da heme oxigenase-1 (HO-1) e da pró-caspase-3 estavam reduzidos nos tecidos renais, enquanto a expressão da caspase-3 clivada mostrou-se elevada. A administração de Tempol na dose de 50 mg/kg mitigou eficazmente essas alterações, indicando que o Tempol atenua a peroxidação lipídica e melhora a injúria renal por meio da via de sinalização PI3K/Akt/Nrf2^6^. Em um modelo murino de lesão por isquemia-reperfusão renal, os níveis renais de progranulina (PGRN) apresentaram redução acentuada. Em contrapartida, camundongos deficientes em PGRN (Grn(–/–)) apresentaram níveis elevados de CREA sérica, aumento da apoptose de células epiteliais tubulares e maior infiltração de neutrófilos e macrófagos no interstício renal, culminando em disfunção renal agravada. A administração de PGRN humano recombinante atenuou as respostas inflamatórias induzidas por hipóxia e a apoptose em células epiteliais do túbulo proximal, um processo associado às respostas imunes mediadas por NOD2. Notavelmente, tanto o pré-tratamento quanto a administração tardia de PGRN humano recombinante protegeram camundongos do tipo selvagem e Grn(-/-) contra lesão por isquemia-reperfusão renal, seja prevenindo a lesão, seja favorecendo sua recuperação, observando-se efeitos protetores comparáveis na IRA induzida por cisplatina^7^.

Estudos demonstraram que agentes nefrotóxicos, incluindo a cisplatina e os meios de contraste, podem desencadear a IRA por meio de vias de sinalização distintas. Especificamente, a cisplatina induz danos ao DNA nas células epiteliais tubulares renais, ativando, assim, a via de sinalização da p53. Essa via modula a expressão de genes envolvidos na parada do ciclo celular e na morte celular programada, culminando no aumento da apoptose. Nas células epiteliais tubulares renais expostas à cisplatina, observa-se uma regulação positiva da expressão da proteína p53, caracterizada por maior expressão do gene pró-apoptótico Bax, redução da expressão do gene antiapoptótico Bcl-2 e aumento da atividade de proteínas executoras da apoptose, como a caspase-3, levando, em última instância, à apoptose^8^. Em rins envelhecidos, a expressão reduzida da α(E)-catenina torna as células epiteliais tubulares renais mais vulneráveis à IRA induzida por medicamentos. Evidências empíricas indicam que a IRA induzida por cisplatina resulta em um aumento de 5,5 vezes na expressão de Fas em células C2 com supressão estável da α(E)-catenina, em comparação com células controle NT-3 não direcionadas. Este processo é acompanhado por um aumento na ativação das caspases-8 e -9, redução na expressão de Bcl-2 e elevação da clivagem de BID e da liberação de citocromo C, indicando uma amplificação da sinalização apoptótica mediada por Fas^8^. A via de sinalização do NF-κB sofre alterações significativas na IRA induzida por cisplatina. No terceiro dia após a administração de cisplatina (25 mg/kg) em camundongos, observaram-se manifestações de IRA, necrose tubular aguda (NTA) e apoptose, juntamente com a ativação da via de sinalização NF-κB no tecido renal. A administração de JSH-23 (40 mg/kg), que modula diretamente a atividade transcricional do NF-κB, melhorou a função renal e reduziu a lesão tubular, conforme evidenciado pela redução da NTA e dos níveis séricos de lipocalina associada à gelatinase neutrofílica (NGAL), embora não tenha exercido impacto significativo na apoptose. Investigações adicionais demonstraram que tanto a cisplatina quanto o JSH-23 podem regular positivamente ou negativamente a expressão de genes associados à via NF-κB, incluindo IL-10, IFN-γ e caspase-1^9^.

Além disso, múltiplas vias de sinalização implicadas nas respostas inflamatórias estão intimamente envolvidas na patogênese da IRA. Verificou-se que mediadores inflamatórios, como o fator de necrose tumoral-α (TNF-α) e a interleucina-1β (IL-1β), apresentavam regulação positiva, com um aumento acentuado da apoptose das células epiteliais tubulares renais^5^. Estudos demonstraram ainda que as respostas inflamatórias mediadas por receptores *Toll-Like* (TLR), incluindo TLR-2, TLR-4, NF-κB p65 nuclear, ASK1 fosforilada, TRAF2 fosforilado, IL-1β, IL-6 e IL-18, encontram-se elevadas neste contexto^10^.

Na IRA induzida por sepse, a glicirrizina (GA) apresenta diversos efeitos terapêuticos, incluindo a atenuação das alterações renais patológicas, a redução dos níveis de BUN e CREA, bem como o aumento das taxas de sobrevida em modelos com ratos. Além disso, a GA inibe a produção de citocinas pró-inflamatórias associadas à via de sinalização do NF-κB, especificamente o TNF-α, a IL-1β e a IL-6. Adicionalmente, diminui a produção de óxido nítrico (NO) e prostaglandina E2 (PGE2) nos tecidos renais, ao mesmo tempo em que promove a expressão da óxido nítrico sintase induzível (iNOS) e da ciclooxigenase-2 (COX-2), reduzindo, assim, a apoptose nas células renais^11^.

Ainda, a via de sinalização da angiogênese-Tie2 desempenha um papel nos mecanismos fisiopatológicos da IRA. Estima-se que a injúria renal aguda isquêmica (IR-AKI) afete entre 2% e 7% dos pacientes hospitalizados. A ativação da via de sinalização da tirosina quinase Tie2, envolvida na angiogênese, demonstrou atenuar a lesão por isquemia-reperfusão (IRI). Estudos indicam que, em modelos murinos de IR-AKI, a fosfatase VE-PTP, que atua como regulador negativo da via da angiogênese-Tie2, apresenta regulação positiva nas células endoteliais renais após a isquemia, enquanto a supressão de seu gene confere proteção contra a IR-AKI. Simultaneamente, a injeção do novo análogo angiogênico Hepta-ANG1 ativa de forma eficaz o Tie2 e protege a função renal em camundongos. A análise de sequenciamento de RNA de célula única identificou assinaturas genéticas específicas do endotélio e o surgimento de uma nova subpopulação endotelial glomerular associada à melhora da função renal^12^. A Tabela 1 apresenta um resumo dos tipos, mecanismos e vias da IRA.

**Tabela 1:** Tipos, mecanismos e vias da IRA

Tipo	Patogênese	Vias de sinalização	Moléculas críticas
IRA pré-renal	1. Hipoperfusão sistêmica → redução do fluxo sanguíneo renal (FSR) → diminuição da taxa de filtração glomerular (TFG) 2. Ativação de mecanismos compensatórios 3. Hipoperfusão persistente evolui para IRA intrínseca	1. Via RAAS – Sistema simpático 2. Sinalização do sistema nervoso	Renina, angiotensina II, aldosterona, norepinefrina e hormônio antidiurético (ADH)
IRA intrínseca	1. **Lesão isquêmica**: disfunção mitocondrial, depleção de ATP, sobrecarga de cálcio celular 2. **Nefrotoxicidade**: dano celular direto, estresse oxidativo, infiltração inflamatória 3. **Obstrução tubular**: formação de cilindros, acúmulo de detritos celulares	1. **Via do estresse oxidativo**: via Nrf2/HO-1 2. **Via inflamatória**: via NF-κB, via JAK/STAT3 3. **Via da apoptose & necroptose**: via MAPK, via PI3K/Akt, cascata de caspase 4. **Via relacionada à fibrose**: via TGF-β/Smad	1. **Estresse oxidativo**: espécies reativas de oxigênio (ROS), fator nuclear eritroide 2 relacionado ao fator 2 (Nrf2), heme oxigenase-1 (HO-1) 2. **Inflamação**: fator nuclear kappa B (NF-κB), fator de necrose tumoral alfa (TNF-α), interleucinas (IL-1β, IL-6, IL-18) 3. **Morte celular**: caspase-3, caspase-8, proteína quinase 1/3 que interage com o receptor (RIPK1/3) 4. **Fibrose e reparo**: fator de crescimento transformador β (TGF-β), Smad2/3, fibronectina, colágeno tipo I
IRA pós-renal	Obstrução do trato urinário → aumento da pressão intratubular → diminuição da pressão de filtração efetiva → redução da TFG Obstrução persistente induz inflamação intersticial renal, atrofia tubular e fibrose renal	1. Via inflamatória do NF-κB 2. Via da fibrose TGF-β/Smad 3. Via MAPK	NF-κB, TNF-α, IL-6, TGF-β, Smad2/3 e inibidor tecidual de metaloproteinases (TIMP-1)

## Marcadores de Vias de Sinalização Para o Diagnóstico de IRA

Os métodos diagnósticos clínicos atuais para a IRA, tais como níveis séricos de CREA e medições do débito urinário, são inadequados para a detecção precoce. Consequentemente, o desenvolvimento de novos biomarcadores baseados em vias de sinalização tornou-se essencial para aprimorar a precisão diagnóstica^13^. A lipocalina associada à gelatinase neutrofílica (NGAL) tem emergido como um biomarcador promissor para IRA. Estudos indicam que os níveis plasmáticos de NGAL estão correlacionados com o risco de desenvolvimento de doença renal crônica (DRC) na população geral. Em um estudo de coorte prospectivo envolvendo 4.660 indivíduos sem DRC, acompanhados por um período de 8,3 anos, 467 participantes desenvolveram DRC de início recente. As concentrações plasmáticas de NGAL mostraram-se significativamente associadas a um risco aumentado de DRC de início recente (razão de risco [HR] 1,35; intervalo de confiança de 95% [IC]: 1,11–1,63; p = 0,002), mesmo após ajuste para variáveis de confusão (HR 1,37; IC 95%: 1,09–1,73; p = 0,007). No entanto, essa associação deixou de ser significativa após ajuste para a taxa de filtração glomerular estimada (TFGe) basal (HR 1,09; IC 95%: 0,86–1,37; p = 0,490)^14^.

Em pacientes com COVID-19, pesquisas identificaram diversos biomarcadores associados a vias de sinalização que se mostram promissoras para o diagnóstico precoce da IRA. Uma revisão abrangente da literatura indica que marcadores de lesão do túbulo proximal e metabólitos do ATP em fase inicial, que refletem distúrbios do metabolismo energético, podem atuar como importantes indicadores clínicos para a detecção da IRA^15^. Simultaneamente, estão em curso avanços em novas tecnologias de detecção de biomarcadores urinários moleculares (MURs), tais como a gama-glutamil transferase (GGT), a alanina aminotransferase (AAP) e a N-acetil-β-D-glucosaminidase (NAG). Em modelos murinos de IRA e DRC induzidas por medicamentos, os MURs não apenas facilitam a detecção precoce da nefrotoxicidade em comparação com os métodos diagnósticos clínicos tradicionais, como também apresentam maior precisão diagnóstica, oferecendo uma nova abordagem para o diagnóstico precoce da IRA^16^. A Tabela 2 apresenta um resumo dos biomarcadores para IRA.

**Tabela 2:** Resumo dos biomarcadores diagnósticos da IRA

Biomarcadores	Vantagens	Desvantagens
CREA sérica	Fácil detecção, baixo custo e ampla disponibilidade	Atraso, baixa especificidade tecidual
BUN	Fácil detecção, baixo custo e ampla disponibilidade	Atraso, baixa especificidade
Débito urinário (DU)	Em tempo real, não invasivo	Baixa especificidade
NGAL	Alta sensibilidade e especificidade	Baixa especificidade tecidual
Molécula de lesão renal-1 (KIM-1)	Alta especificidade tecidual	Menor sensibilidade, não adequado para triagem de rotina
NAG	Alta sensibilidade	Baixa especificidade
TIMP-2·IGFBP7	Detecção precoce, alta especificidade	Custo elevado, não adequado para triagem de rotina
**IL-18**	Alta sensibilidade	Baixa especificidade

## Alvos das Vias de Sinalização Para O Tratamento da IRA

O manejo da IRA ainda enfrenta desafios significativos; porém, estratégias terapêuticas direcionadas a vias de sinalização específicas representam uma abordagem promissora para a melhoria dos desfechos na IRA^17^. Estudos indicam que a zingerona pode atenuar a IRA induzida por lipopolissacarídeo (LPS) ao inibir a via de sinalização inflamatória TLR4/NF-κB. Em modelos murinos de IRA induzida por LPS, a administração de zingerona resultou em uma redução dose-dependente dos níveis elevados de BUN, CREA e citocinas inflamatórias, tais como TNF-α, IL-6 e IL-1β, juntamente com uma diminuição das alterações histopatológicas renais. Esses achados sugerem que a zingerona confere efeitos protetores contra a IRA induzida por LPS por meio da inibição dessa via de sinalização^18^.

Na lesão por isquemia-reperfusão renal, o EZH2 (*enhancer of zeste homolog 2*) desempenha um papel fundamental na patogênese da IRA, ao modular a via de sinalização p38. A administração do inibidor seletivo de EZH2, 3-deazaneplanocina A (DZNeP), em camundongos atenuou a disfunção renal e os danos tubulares pós-isquemia-reperfusão, reduziu a apoptose e a ativação da caspase-3 e inibiu o recrutamento de células T CD3+ e células F4/80+, bem como a produção de mediadores inflamatórios, incluindo TNF-α, proteína quimioatraente de monócitos-1 (MCP-1), IL-6 e IL-18. Em experimentos celulares, o tratamento com DZNeP ou a supressão de EZH2 resultou na redução da apoptose em células HK-2 submetidas à hipóxia-reoxigenação. Esse achado indica que o direcionamento da via de sinalização EZH2/p38 pode representar uma nova estratégia para a proteção dos tecidos renais contra a IRA induzida por isquemia-reperfusão^19^.

Paralelamente, determinados agentes farmacológicos demonstram potencial terapêutico na IRA ao modularem diferentes vias de sinalização. Por exemplo, demonstrou-se que a administração de lítio melhora significativamente a função renal em modelos com ratos, conforme evidenciado por escores reduzidos de injúria renal, níveis diminuídos de creatina fosfoquinase (CPK), menor infiltração de macrófagos e menor expressão renal de NF-κB e caspases. Além disso, o tratamento com lítio aumenta os níveis da superóxido dismutase de manganês (MnSOD), uma enzima antioxidante. Esses efeitos terapêuticos são atribuídos, principalmente, à inibição da glicogênio sintase quinase 3β (GSK3β), contribuindo, assim, para a atenuação da IRA associada à rabdomiólise^20^.

## Pontos Críticos na Investigação da via de Sinalização da IRA

A via de sinalização da proteína morfogenética óssea 7 (BMP-7) tem sido um importante foco de pesquisa desde há muito tempo. A BMP-7 desempenha um papel fundamental no desenvolvimento renal e na reparação de lesões, tendo seu potencial terapêutico em modelos de doença renal crônica sido reconhecido há quase duas décadas. Investigações recentes revelaram que a BMP-7 pode mitigar o dano renal na IRA por meio de diversos mecanismos, incluindo a regulação do metabolismo da matriz extracelular, a supressão das respostas inflamatórias e a indução da apoptose. Estudos em modelos animais demonstraram que a administração de BMP-7 recombinante melhora a função renal e reduz os danos patológicos, sugerindo uma estratégia terapêutica biológica promissora para o tratamento da IRA^21^.

As vias de sinalização relacionadas à morte celular não apoptótica permanecem um foco central de pesquisa. Na IRA induzida por sepse, a proteína quinase 3 que interage com receptores (RIPK3) demonstrou agravar a lesão tubular renal ao promover necroptose, estresse oxidativo e disfunção mitocondrial. Estudos adicionais identificaram a RIPK3 e a NADPH oxidase-4 (NOX4) como fatores críticos na lesão tubular renal *in vivo*, oferecendo, assim, novos alvos terapêuticos para a IRA^22^.

Análises transcriptômicas proporcionaram novos *insights* sobre as vias de sinalização associadas à IRA. Por meio do perfil transcriptômico de células epiteliais tubulares renais danificadas, pesquisadores identificaram múltiplas vias de sinalização e redes de regulação gênica que desempenham papéis na plasticidade epitelial, no reparo tecidual e na fibrose. O estudo demonstrou que células epiteliais tubulares renais lesionadas tentam se reparar por meio da reexpressão de determinados genes nefrogênicos; no entanto, esse processo se desvia das vias normais de desenvolvimento, resultando em um mecanismo de resposta regenerativa exclusivo. Esses achados aprofundam a compreensão dos mecanismos moleculares subjacentes à IRA e fornecem uma base teórica para o desenvolvimento de estratégias terapêuticas inovadoras^23^. Por meio do uso de técnicas como a análise de rede de coexpressão gênica ponderada (WGCNA), pesquisadores identificaram redes críticas de miRNA-mRNA associadas à IRA. Em estudos centrados na IRA pós-transplante, a análise WGCNA de dois conjuntos de dados (GSE53771 e GSE53769) revelou inúmeras interações entre mRNA e miRNA. Análises adicionais utilizando o miRDIP v4.1 previram módulos-chave de interação miRNA-mRNA, construindo assim uma rede regulatória abrangente. Em particular, nós como miR-203a-3p, miR-205-5p e ERBB4 demonstraram conectividade significativa, indicando seus papéis fundamentais no desenvolvimento de IRA pós-transplante. Paralelamente, análises realizadas com base em *Gene Ontology* (GO) e nas vias da *Kyoto Encyclopedia of Genes and Genomes* (KEGG) revelaram que essa rede abrange predominantemente funções e vias de sinalização relacionadas aos rins, incluindo PI3K-Akt, HIF-1, Ras e MAPK. Esses achados fornecem evidências essenciais para a identificação de novos biomarcadores ou agentes terapêuticos, aprimorando assim as estratégias de predição e intervenção precoces para a IRA^24^.

Os biomarcadores associados à metilação do DNA têm suscitado um interesse acadêmico cada vez maior. Por meio da análise da metilação do DNA no sangue total de participantes do *Taiwan Biobank*, pesquisadores identificaram genes específicos e vias de sinalização associados ao envelhecimento renal e à deterioração da função renal. Entre os 1.587 participantes avaliados, 187 apresentaram taxas aceleradas de declínio da TFGe. Ao comparar os padrões de metilação entre participantes com diferentes taxas de declínio da TFGe e entre faixas etárias distintas, foram identificados genes com hipermetilação comum, como DNMT3A e GGACT, bem como genes hipometilados, incluindo ARL6IP5, CYB5D1, BCL6, RPRD2, ZNF451 e MIAT. Além disso, verificou-se que o estado de metilação de vias de sinalização, como autofagia, p38 MAPK e sirtuínas, está associado ao envelhecimento e à disfunção renal. Esses achados contribuem para o desenvolvimento de novos biomarcadores para a identificação de populações de alto risco e oferecem *insights* sobre potenciais alvos terapêuticos^25^.

Na pesquisa sobre fibrose renal, o STAT3 desempenha papel crucial. Estudos utilizando camundongos *knockout* para STAT3 mediado por Foxd1, CRISPR e inibidores específicos demonstraram que a fosforilação dessa proteína ocorre nas células epiteliais tubulares renais durante a IRA, estendendo-se às células intersticiais na fibrose. A deficiência de STAT3 mediada por Foxd1 conferiu proteção aos camundongos contra a fibrose induzida por ácido fólico e ácido aristolóquico. Esse fator intensifica as respostas inflamatórias e a diferenciação de pericitos em miofibroblastos, promovendo migração e sinalização fibrogênica em células semelhantes a pericitos submetidas à edição genômica. Sua inibição reduz o desprendimento celular, a migração e a sinalização fibrogênica. Além disso, o STAT3 se liga ao promotor do colágeno tipo I alfa1 nos rins e nas células de camundongos. Este estudo identifica o STAT3 como um novo promotor da fibrose renal, sugerindo-o como um potencial alvo para terapia gênica^26^.

Além disso, a LRP5, um correceptor transmembranar multifuncional, desempenha um papel fundamental na IRA. Essa proteína atua principalmente por meio da via de sinalização Wnt/β-catenina, ao mesmo tempo em que modula a função renal por vias não clássicas, como AKT/P21 e TGF-β/Smad. Embora a LRP5 demonstre efeitos protetores no contexto da IRA, ela contribui para a progressão da doença em condições patológicas crônicas, incluindo fibrose tubulointersticial renal, doença renal policística e aterosclerose, ao ativar vias fibrogênicas e inflamatórias. Utilizando a técnica de *knockout* por CRISPR/Cas9 e outras tecnologias de edição gênica, os pesquisadores podem realizar investigações abrangentes sobre os mecanismos da LRP5 na IRA e em doenças relacionadas, proporcionando, assim, uma base teórica para o desenvolvimento de estratégias terapêuticas baseadas na LRP5^27^.

## Pontos Controversos no Estudo das Vias de Sinalização da IRA

Na investigação das vias de sinalização da IRA, foram alcançados avanços significativos; entretanto, diversas questões controversas persistem, demandando estudos adicionais^8^. Os mecanismos de morte celular e suas vias de sinalização associadas na IRA permanecem como objetos de debate. Por exemplo, estudos sobre o envelhecimento dos rins, os quais são mais suscetíveis à IRA induzida por medicamentos, demonstraram que a expressão reduzida da α(E)-catenina potencializa a via de apoptose mediada por Fas. No entanto, papéis e interações precisos de outros mecanismos de morte celular, como a necroptose e a ferroptose, neste contexto ainda não são totalmente compreendidos. Alguns estudos indicam que a necroptose pode atuar em sinergia com a apoptose para intensificar a injúria renal em determinadas condições, enquanto outros sugerem que esses processos podem operar de forma independente, em diversos estágios ou em resposta a diferentes estímulos^8^.

Os mecanismos regulatórios que regem as vias de sinalização inflamatória também permanecem em debate. A via de sinalização do NF-κB constitui um exemplo relevante, uma vez que sua ativação e inibição apresentam efeitos variáveis sobre a injúria renal em diferentes modelos e estudos de IRA. Em algumas investigações, a inibição da sinalização do NF-κB demonstrou mitigar a inflamação e o dano renal. Por outro lado, a ativação dessa via parece contribuir para os processos de reparo renal. Por exemplo, durante os estágios iniciais da lesão por isquemia-reperfusão renal, a ativação moderada do NF-κB pode facilitar o início das respostas imunes necessárias para a eliminação de células danificadas e patógenos. No entanto, uma ativação excessiva pode resultar em inflamação descontrolada e danos teciduais agravados. Portanto, a regulação precisa da via de sinalização do NF-κB é essencial para alcançar desfechos terapêuticos ideais, o que requer investigações adicionais^9^.

Ademais, as interações entre as diversas vias de sinalização e suas alterações dinâmicas ao longo dos diferentes estágios da IRA ainda constituem tema de debate. Notavelmente, as vias de sinalização PI3K-Akt e mTOR são essenciais para a IRA, demonstrando complexas relações de regulação mútua. Apesar disso, os mecanismos regulatórios específicos e seus papéis predominantes nas distintas fases da IRA, tais como as fases de injúria e reparo, ainda não são totalmente compreendidos. Alguns estudos sugerem que a via PI3K-Akt está envolvida principalmente na sobrevida celular e nos processos antiapoptóticos durante a fase inicial de lesão, enquanto a via mTOR pode desempenhar um papel mais central na proliferação celular e no reparo tecidual durante a fase de reparo. No entanto, esses achados requerem investigações adicionais para validação e refinamento^28^.

## Perspectivas Futuras das Vias de Sinalização da IRA

Espera-se que a pesquisa sobre as vias de sinalização da IRA registre avanços em diversas frentes, podendo levar a descobertas significativas na compreensão de sua patogênese e no desenvolvimento de estratégias terapêuticas eficazes^29^. O papel do sulfeto de hidrogênio (H_2_S) na fisiologia e na patologia renal merece uma investigação mais aprofundada. Como molécula de sinalização gasosa, o H_2_S desempenha um papel regulador em processos fisiológicos renais fundamentais, incluindo a filtração glomerular e a reabsorção de sódio. Nas doenças renais, os efeitos do H_2_S são complexos e dependentes do contexto. Por exemplo, em condições como a lesão por isquemia-reperfusão e a nefropatia diabética, demonstrou-se que o H_2_S demonstrou atenuar os danos renais. Por outro lado, seu papel na nefrotoxicidade induzida por cisplatina ainda não está totalmente elucidado. Espera-se que pesquisas futuras examinem de forma abrangente a influência desse gasotransmissor na fisiologia renal, elucidem seus mecanismos em doenças renais relacionadas à inflamação e à toxicidade, avaliem seu potencial como alvo terapêutico em distúrbios renais específicos e desenvolvam intervenções terapêuticas baseadas no H_2_S^29^.

A investigação sobre o metabolismo lipídico é essencial não apenas para o fornecimento de energia nos rins, mas também para a formação de biofilmes renais e o desenvolvimento do microambiente renal. Metabólitos resultantes do metabolismo lipídico influenciam significativamente os processos celulares, incluindo a proliferação, a diferenciação e a apoptose, por meio de vias de transdução de sinal. Embora as pesquisas atuais abordem predominantemente as anomalias do metabolismo lipídico na DRC, os estudos sobre distúrbios do metabolismo lipídico associados à IRA ainda são relativamente escassos. Pesquisas futuras poderão investigar os metabólitos lipídicos como potenciais biomarcadores para o diagnóstico precoce e a classificação da IRA, bem como avaliar o impacto da regulação do metabolismo lipídico na progressão da IRA. Tais esforços poderão revelar novos alvos terapêuticos e estratégias de intervenção para o manejo da IRA^30^.

Ao mesmo tempo, os avanços contínuos na transcriptômica de célula única e nas tecnologias multi-ômicas têm aprimorado nossa capacidade de identificar com precisão as alterações nas vias de sinalização em diferentes tipos de células durante a IRA. Por meio da aplicação da análise transcriptômica de célula única em tecidos renais, os pesquisadores podem obter uma compreensão mais aprofundada dos distintos padrões de ativação das vias de sinalização em diversas populações celulares, incluindo células epiteliais tubulares renais, células endoteliais e células do sistema imunológico, durante a IRA. Essa abordagem também elucida os mecanismos da sinalização intercelular. Esses subsídios são fundamentais para desvendar a complexa patogênese da IRA e estabelecem as bases para o desenvolvimento de estratégias terapêuticas de precisão que tenham como alvo tipos específicos de células e vias de sinalização^31^.

A investigação sobre as vias de sinalização da IRA enfrenta inúmeros desafios, mas também apresenta oportunidades significativas para avanços contínuos na área^32^. A complexa patogênese da IRA envolve interações entre múltiplas vias de sinalização e diversos tipos de células, o que dificulta a compreensão abrangente de seus processos patológicos (Figura 1). Na IRA induzida por lesão por isquemia-reperfusão, as vias de sinalização relacionadas à inflamação são ativadas, e também ocorrem alterações em vias associadas a processos celulares, como apoptose e autofagia. Essas vias de sinalização apresentam relações inter-regulatórias complexas, tornando a análise abrangente e precisa de suas alterações dinâmicas e interações um desafio significativo^32^. A tradução dos achados da pesquisa básica em aplicações clínicas permanece desafiadora. Certos inibidores ou ativadores de vias de sinalização que apresentam efeitos renoprotetores promissores em modelos animais muitas vezes não alcançam os resultados esperados em ensaios clínicos com seres humanos, principalmente devido a questões farmacocinéticas e de segurança. O aprimoramento da eficiência da tradução clínica dos achados da pesquisa básica constitui uma questão urgente que requer atenção. Ainda assim, a pesquisa sobre as vias de sinalização da IRA oferece inúmeras oportunidades. O advento de tecnologias avançadas, como a edição gênica (por exemplo, CRISPR/Cas9), o sequenciamento de células únicas e imagens de alta resolução, proporcionou aos pesquisadores ferramentas poderosas para a realização de investigações detalhadas sobre essas vias de sinalização. As tecnologias de edição gênica facilitam a análise precisa dos papéis de genes específicos nessas vias, enquanto o sequenciamento de células únicas esclarece as alterações dessas vias em diferentes tipos celulares durante a IRA. Além disso, as imagens de alta resolução permitem capturar a dinâmica molecular em tempo real nos tecidos renais. Em conjunto, esses avanços tecnológicos prometem revolucionar a pesquisa sobre as vias de sinalização da IRA^26^.

**Figura 1:** Descrição das múltiplas vias de sinalização interligadas envolvidas na patogênese da IRA.

## Data Availability

Nenhum dado novo foi gerado ou analisado neste estudo.
